# Early prolonged ambulatory cardiac monitoring in stroke (EPACS): an open-label randomised controlled trial

**DOI:** 10.1186/s40001-019-0383-8

**Published:** 2019-07-26

**Authors:** Amit Kaura, Laszlo Sztriha, Fong Kum Chan, John Aeron-Thomas, Nicholas Gall, Bartlomiej Piechowski-Jozwiak, James T. Teo

**Affiliations:** 1 King’s College London NHS Foundation Trust, King’s College Hospital, Denmark Hill, London, SE5 9RS UK; 2 Imperial College Healthcare NHS Trust , Hammersmith Hospital, Du Cane Road, London, W12 0HS UK

**Keywords:** Atrial fibrillation, Cardiac monitoring, Electrocardiography, Ischaemic stroke, Medical devices

## Abstract

**Background:**

Cardioembolism in paroxysmal atrial fibrillation (PAF) is a preventable cause of transient ischaemic attack (TIA) or ischaemic stroke; however, due to its transient nature, a short-duration Holter monitor may miss a significant proportion of events.

**Methods:**

We conducted an open-label randomised controlled trial of cardiac monitoring after a TIA or ischaemic stroke comparing a 14-day ECG monitoring patch (Zio^®^ Patch, iRhythm Technologies) with short-duration Holter monitoring for the detection of PAF. The primary outcome was the detection of one or more episodes of ECG-documented PAF lasting at least 30 s within 90 days in each of the study arms. A budget impact analysis from the healthcare perspective was performed.

**Results:**

From February 2016 through February 2017, 43 (76.8%) of the 56 patients assigned to the patch-based monitoring group and 47 (78.3%) of the 60 patients assigned to short-duration Holter monitoring group had successful monitor placement with 90 days of follow-up. Of the 26 protocol failures between the two groups, 23 (88.5%) were due to patient refusal for outpatient short-duration ECG monitor placement, whilst only 1 (3.8%) was due unsuccessful ZioPatch placement. The rate of detection of PAF at 90 days was 16.3% in the patch-based monitoring group (seven patients) compared to 2.1% in the short-duration Holter monitoring group (1 patient), with an odds ratio of 8.9 (95% CI 1.1–76.0; *P* = 0.026). An economic model demonstrated that implementation of the Zio Patch service would result in 10.8 more strokes avoided per year compared to current practice with Holter monitoring with an associated yearly saving in direct medical costs of £113,630, increasing to £162,491 over 5 years.

**Conclusions:**

Early, prolonged, patch-based monitoring after an index stroke or TIA is superior to short-duration Holter monitoring in the detection of PAF and likely cost-effective for preventing recurrent strokes.

*Trial registration* http://www.isrctn.com. Unique identifier: ISRCTN 50253271. Registered 21 January 2016

**Electronic supplementary material:**

The online version of this article (10.1186/s40001-019-0383-8) contains supplementary material, which is available to authorized users.

## Background

A frequent cause of ischaemic stroke or transient ischaemic attack (TIA) is due to cardioembolism in atrial fibrillation (AF), but up to 40% of ischaemic strokes are classified as cryptogenic, with no cause identified after routine evaluation [1, 2]. This is because AF is often paroxysmal (paroxysmal atrial fibrillation, PAF) and a single 12-lead ECG or even 24-h Holter ECG monitoring may miss a significant proportion of patients with PAF [3]. While stroke guidelines from both North America and the UK suggest that more prolonged cardiac rhythm monitoring may be indicated, there is currently no consensus on the recommended duration of ECG monitoring [4, 5].

Available approaches for ECG monitoring beyond 24 h have their limitations. While continuous bedside ECG monitoring during an approximately 3-day inpatient stay on the stroke unit has a significantly higher sensitivity in detecting PAF than 24-h Holter monitoring (7.7% vs 2.8%) [6], this approach is not suitable for outpatients who are ambulant immediately following their stroke. While systems for recording beyond 3–7 days have demonstrated even higher detection rates of PAF [7, 8], they have significant limitations: event-triggered loop recorders are cumbersome while implanted loop recorders require a minor surgical procedure. There is therefore a need for a patient-friendly, long-duration, ECG monitoring system for patients with cryptogenic stroke or TIA. The ZioPatch^®^ (iRhthym Technologies, USA) is a novel, adhesive cardiac monitoring patch which provides an alternative method for prolonged ECG monitoring for the detection of PAF [9–11]. This waterproof patch is applied non-invasively to the anterior chest wall for continuous monitoring for up to 14 days without requiring any complex setup. The ECG trace uses the Zio XT algorithmic support to highlight areas for human interpretation.

We conducted a pragmatic randomised controlled trial of cardiac monitoring for the detection of PAF, in patients with cryptogenic stroke or TIA, using the patch-based monitor or a standard short-duration Holter monitor. We hypothesised that early prolonged ambulatory cardiac monitoring would enhance the detection of PAF in patients who would be candidates for anticoagulation therapy for secondary stroke prevention.

## Methods

### Study design

The early prolonged ambulatory cardiac monitoring in stroke (EPACS) study was a prospective randomised open-blinded endpoint trial comparing the efficiency of PAF detection with the 14-day patch-based monitor versus conventional medical therapy of short-duration Holter ECG in patients with cryptogenic ischaemic stroke or TIA early after the index event. With a sample size of 120, the study was powered at 90% (beta set at 0.10), based on a conservative effect size from previous studies (EMBRACE [8] and CRYSTAL-AF [7]). A single interim analysis at a sample size of 40 participants was performed using O’Brien-Fleming stopping boundaries to maintain an overall alpha level of 0.05.

**Fig. 1:**
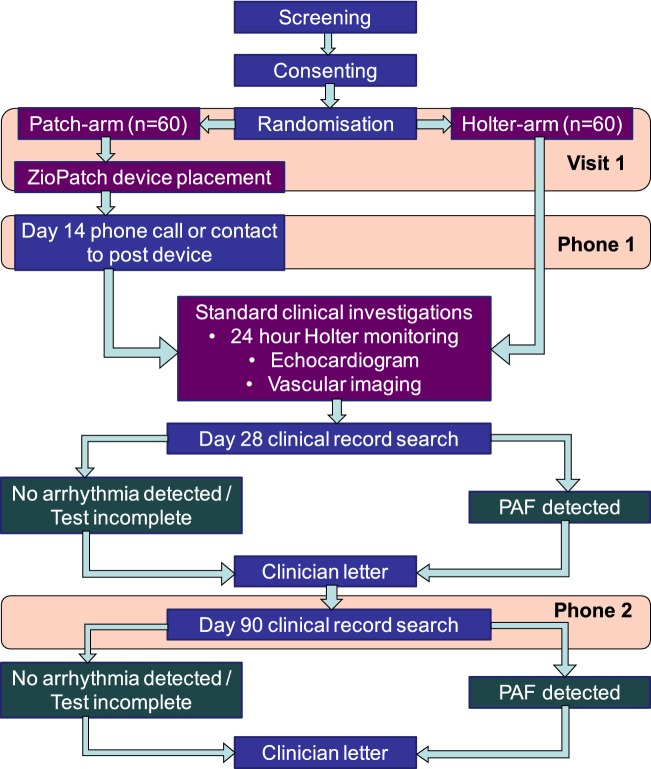
Flowchart of trial protocol. *PAF* paroxysmal atrial fibrillation

The study protocol is summarised in Fig. 1. Patients were randomly assigned in a 1:1 ratio to one of the two ECG monitoring strategies using a computerised block randomisation generator provided externally by the King’s Clinical Trials Unit, stratified for age, gender and history of hypertension. Patients were enrolled from King’s College Hospital NHS Foundation Trust, UK, across two sites, King’s College Hospital, an urban teaching hospital, and Princess Royal University Hospital, a suburban district general hospital.

All patients were provided with verbal and written information about the trial, and informed written consent was obtained before enrolment. The trial was registered with The National Research Ethics Service and the local Research and Development Committee approved the study protocol. The Medicines and Healthcare products Regulatory Agency verified EPACS as a post-marketing study which did not require specific authorisation. The trial was registered on the International Standard Randomised Controlled Trial Number Register (ISRCTN 50253271).

The trial was conducted in accordance with the principles of the Declaration of Helsinki and the guidelines for Good Clinical Practice (EMA/CPMP/CIH/135/1995).

The funder (Bristol–Meyers–Squibb) provided financial support for this project and had no influence in the conduct of the study or the drafting of the analysis. The manufacturer of the ZioPatch cardiac monitor (iRhythm Technologies, USA) was not involved in the study design, conduct of trial or in the drafting of this paper; iRhythm provided support for inventory management and remote data management of devices.

### Patient eligibility

Eligible patients were 18 years of age or older and were diagnosed with having had an ischaemic non-lacunar stroke or TIA within the past 72 h by a stroke physician or neurologist. Patients with a TIA were enrolled only if there were cortical symptoms of hemianopia or dysphasia at presentation or if their diffusion-weighted cerebral MRI scan was positive in a non-lacunar distribution. The main exclusion criteria were a history of AF or atrial flutter, carotid stenosis > 50%, a pre-existing indication or contraindication for permanent anticoagulation therapy or those unable to provide informed consent.

### ECG monitoring strategies

Patients assigned to the conventional medical therapy arm received current medical therapy of ambulatory Holter monitoring only (duration determined by treating physician, which was usually 24 h), either arranged as an inpatient or outpatient depending on the anticipated duration of inpatient stay as per hospital protocol. Patients assigned to the patch-based monitoring arm had the patch applied to the anterior chest wall with the device kept in-situ for 14-days continuously, removed only for short intervals, such as during MRI imaging. Patients discharged from hospital prior to day 14 were discharged with the patch in-situ. On day 14, patients received a phone call reminder to return the device in a postage-paid envelope (Fig. 1). The patch was directly received by the manufacturer and the data retrieved and processed by the company’s Zio ECG Utilisation Service. The summary report was subsequently accessed via a secure website and the reports reviewed by a cardiologist for report verification following review of the rhythm strips provided. Patients randomised to the patch-based monitoring arm also had standard practice of short-duration Holter monitoring. The Lifecard CF Holter Recorder (Spacelabs Healthcare, USA) was used for the Holter ECG. All ECG traces supplied from the short-duration Holter monitor and patch-based monitor were assessed by an experienced cardiologist; the patch-based monitor used an automated algorithmic support to highlight areas for diagnostic interpretation. Patients in both study arms were followed up for 90 days, without direct contact by the research team.

### Follow-up and clinical outcomes

At day 28 and at 90, we conducted an electronic hospital medical records data search and phone call to the patient’s general practitioner in the community to collect endpoint data (Fig. 1). The primary outcome was the detection of one or more episodes of ECG-documented PAF lasting at least 30 s within 90 days in each of the study arms (inter-subject comparison). This included PAF documented on the ECG monitoring strategies or detected incidentally during usual clinical practice, such as during echocardiography.

Secondary outcomes included PAF lasting at least 30 s within 28 days in each of the study arms and PAF lasting at least 30 s detected on the patch-based monitoring or short-duration Holter monitor within 90 days in patients who underwent both ECG monitoring strategies (intra-subject comparison). Other secondary outcomes included anticoagulation therapy use at day 90, the proportion of patients with ischaemic stroke or TIA at day 90, mortality at day 90 and time-to-reporting of ECG monitoring. Decisions on patient management (including anticoagulation) were made by the patient’s responsible physician and not the research team.

### Economic evaluation

Formal cost-effectiveness assessment of new diagnostic devices is desirable. However, the current study yields insufficient information on clinical outcomes to support a cost-utility model, given the limited 90-day follow-up period. This process will need to be undertaken as more data become available in the future. As an indicative exercise, a budget impact analysis from the healthcare perspective was carried out by Imperial College Health Partners to assess the theoretical economic implications of the Zio Patch service versus Holter monitoring. Based on the AF detection rates found in this study, Hospital Episode Statistics data for the incidence of stroke and TIA (October 2016–September 2017) [12] and NHS reference costs [13], the cost-effectiveness of the ZioPatch service versus Holter monitoring at King’s College Hospital NHS Foundation Trust was calculated. The Sentinel Stroke National Audit Programme estimate of £13,452 was used as the mean year 1 direct medical cost of a stroke [14]. All probability and cost assumptions made for the economic model can be reviewed in Additional file 1.

### Statistical analyses

Per-protocol analyses were performed for all comparisons of outcome as intention-to-treat was not balanced due to the high drop-out rate for Holter ECG’s. Categorical variables were compared using the Fisher’s Exact test. The *t* test was used to compare continuous variables that were approximately normally distributed and the Wilcoxon Mann–Whitney Rank-Sum test was used for continuous variables that were not normally distributed. Statistical significance was set at *P *< 0.05 (2-sided). SPSS (version 21.0, SPSS Inc., Chicago, USA) was used for the statistical analyses.

## Results

### Study population

**Fig. 2:**
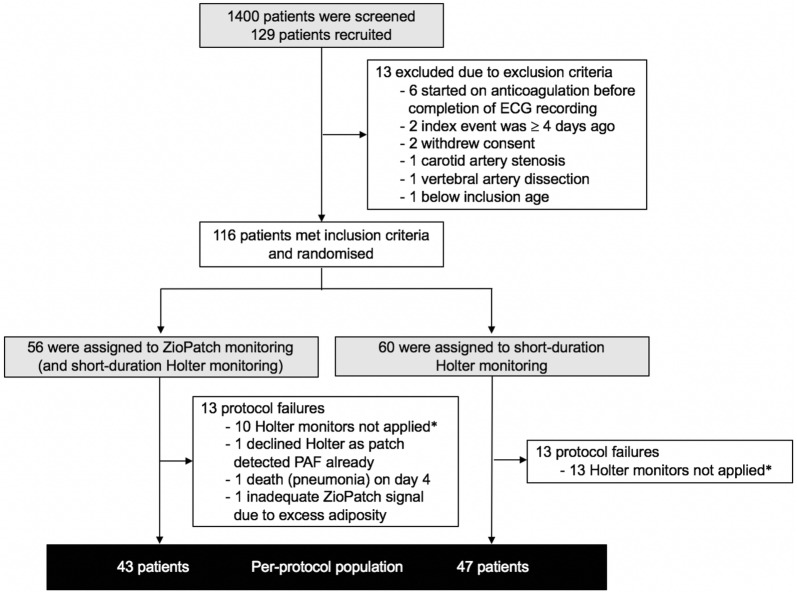
Flowchart of enrolment, randomisation and follow-up of study participants. *ECG* electrocardiography, *PAF* paroxysmal atrial fibrillation. *Patient refusal for outpatient short-duration ECG monitor placement

From February 2016, through to February 2017, a total of 129 patients were recruited and 116 were randomly assigned to either the patch-based monitoring group (56 patients) or the short-duration Holter monitoring group (60 patients) (Fig. 2). The mean (± SD) time between the index event and randomisation was 2.0 ± 1.2 days. Of the 56 patients assigned to the ZioPatch group, 43 (76.8%) completed 90 days of follow-up with successful patch-based monitoring and short-duration Holter monitor recordings. Forty-seven (78.3%) of the 60 patients assigned to short-duration Holter monitoring had successful monitor placement with 90 days of follow-up. Of the 26 protocol failures between the two groups, 23 (88.5%) were due to patient refusal for outpatient short-duration ECG monitor placement, whilst only 1 (3.8%) was due unsuccessful ZioPatch placement. The mean time from stroke or TIA to device placement was 2.1 ± 1.2 days and 38.9 ± 33.6 days with the ZioPatch and short-duration monitor, respectively. The mean wear time for the 14-day patch-based monitor and short-duration Holter monitor was 283.8 ± 88.7 h and 25.0 ± 25.0 h, respectively. Patch-based monitoring was completed successfully in 54 out of 56 patients (96.4%) with the majority completing the full 14-days of recording.

Baseline characteristics of the randomly assigned patients are shown in Table 1. The mean age was 70.4 ± 13.2 years, 38.9% of patients were women, and 85.6% of index events were classified as a stroke. The mean CHADS_2_VASc_2_ score of all patients was 4.3 ± 1.0 points. None of the patients had previously received anticoagulation therapy or had a previous diagnosis of AF.

**Table 1:** Baseline characteristics of the study participants

Characteristic	Patch-based monitoring (ACTIVE) (*n *= 43)	Short-duration Holter (CONTROL) (*n *= 47)	*P* value
Age, year	70.7 ± 12.6	70.0 ± 13.9	0.82
Sex, no. (%)			
Male	26 (60.5)	29 (61.7)	1.00
Female	17 (39.5)	18 (38.3)	
Race or ethnic group, no. (%)			
Asian	1 (2.3)	2 (4.3)	0.80
Black	11 (25.6)	10 (21.3)	
White	31 (72.1)	35 (74.5)	
Recruitment site, no. (%)			
King’s College Hospital	12 (27.9)	22 (46.8)	0.08
Princess Royal University Hospital	31 (72.1)	25 (53.2)	
Index event, no. (%)			
Stroke	35 (81.4)	43 (91.5)	0.22
TIA	8 (18.6)	4 (8.5)	
Prior stroke or TIA, no. (%)	12 (27.9)	7 (14.9)	0.20
Score on NIH stroke scale (stroke patients)^a^	2.3 ± 3.7	2.1 ± 2.6	0.90
Score on ABCD^2^ (TIA patients), no.	4.1 ± 0.6	4.2 ± 0.8	0.87
Hypertension, no. (%)	26 (60.5)	30 (63.8)	0.83
Diabetes, no. (%)	10 (23.3)	10 (21.3)	1.00
CHADS_2_VASc_2_ score, no.^b^	4.4 ± 1.2	4.3 ± 1.0	0.61
Ischaemic heart disease, no. (%)	8 (18.6)	5 (10.6)	0.37
Hypercholesterolaemia, no. (%)	17 (39.5)	17 (36.2)	0.83
Smoking status, no. (%)			
Ex-smoker	12 (27.9)	5 (10.6)	0.27
Current smoker	6 (14)	10 (21.3)	
Use of antiplatelet, no. (%)^c^			
None	30 (69.8)	33 (70.2)	0.26
Aspirin	5 (11.6)	10 (21.3)	
Clopidogrel	6 (14.0)	4 (8.5)	
Aspirin and Clopidogrel	2 (4.7)	0 (0)	
No. of days from index event to randomisation	2.0 ± 1.2	1.9 ± 1.1	0.60

Plus–minus values are mean ± SD. *P* values were calculated with the use of Student’s *t* test, Wilcoxon Mann–Whitney Rank-Sum test or Fisher’s exact test, as appropriate. TIA denotes transient ischaemic attack

*NIH* National Institutes of Health, *TIA* transient ischaemic attack

^a^Scores on the National Institutes of Health Stroke Scale range from 0 to 42, with higher scores indicating more severe neurologic deficits. The score was not reported for three patients in the patch-based monitoring (ACTIVE) group and four patients in the 24-h Holter monitoring group

^b^Scores on the CHADS_2_VASc_2_ risk assessment range from 0 to 6, with higher scores indicating a greater risk of stroke

^c^Antiplatelet therapy before the index stroke or TIA

### Primary endpoint

The patch-based monitoring strategy was superior to short-duration Holter monitoring for the detection of PAF lasting 30 s or longer—the rate of detection of PAF at 90 days was 16.3% among patients assigned to the ZioPatch patch-based monitoring group (seven patients), as compared to 2.1% among patients assigned to the short-duration Holter monitoring group (one patient), with an odds ratio of 8.9 (95% confidence interval (CI) 1.1–76.0; *P *= 0.026) (Table 2). One of the seven patients who had PAF in the patch-based monitoring group had PAF detected on the short-duration Holter monitor recording alone.

**Table 2:** Detection atrial fibrillation and the effect of treatment in the two monitoring groups

Outcome	Patch-based monitoring (ACTIVE) (*n *= 43)	Short-duration Holter (CONTROL) (*n *= 47)	*P* value	Odds ratio (95% CI)
	Number (percentage)			
Primary outcome				
Detection of PAF with duration ≥ 30 s at 90 days (inter-subject comparison)	7 (16.3)	1 (2.1)	0.026	8.9 (1.1–76.0)
Secondary outcomes				
Detection of PAF with duration ≥ 30 s at 28 days	6 (14.0)	1 (2.1)	0.051	7.5 (0.9–64.7)
Anticoagulation therapy use at 90 days	7 (16.3)	1 (2.1)	0.026	8.9 (1.1–76.0)
Second ischaemic stroke or TIA at 90 days	1 (2.3)	1 (2.1)	1.00	1.1 (0.1–18.1)
Mortality at 90 days	1 (2.3)	0 (0)	0.48	–

*PAF* paroxysmal atrial fibrillation, *TIA* transient ischaemic attack

### Secondary endpoints

The rate of detection of PAF at 28 days was 14.0% (6 patients) in the patch-based monitoring group, as compared to 2.1% (1 patient) in the short-duration Holter monitoring group (odds ratio 7.5; 95% CI 0.9–64.7; *P *= 0.05) (Table 2). In the patch-based monitoring group, the detection of PAF with duration ≥ 30 s at 90 days in patients who underwent both ECG monitoring strategies was 16.3% (7 patients) using patch-based monitoring, as compared to 4.7% (2 patients) using 24-h Holter monitoring (odds ratio 4.0; 95% CI 0.8–20.4; *P* = 0.16). All patients who had newly diagnosed PAF were commenced on anticoagulation therapy by day 90. No short-term anticoagulation therapy-related adverse events were recorded by day 90. There was no difference in the rate of recurrent ischaemic stroke or TIA (patch-based monitoring vs short-duration Holter; 1 (2.3%) vs 1 (2.1%); *P *= 1.00) or mortality [1 (2.3%) vs 0 (0%); *P *= 0.48] at 90 days.

### Characteristics of patients with atrial fibrillation

By 90 days of follow-up, among patients in the patch-based monitoring group with PAF detected, the mean time spent in AF in a single day was 4.2 ± 6.2 h and the mean value for the maximum time spent in AF was 44.6 ± 78.6 h. In the patch-based monitoring group, patients with PAF were significantly older than patients with no evidence of PAF, (PAF vs no PAF; 77.4 ± 6 vs 68.6 ± 14.6; *P *= 0.02) (Table 3). Patients with PAF also had a history of ischaemic heart disease (57.1% vs 11.1%; *P *= 0.02), had a higher CHADS_2_VASc_2_ score (5.0 ± 0.6 vs 4.3 ± 1.2; *P *= 0.03), had left atrial enlargement (85.7% vs 39.2%; *P *= 0.04) and had episodes of non-sustained ventricular tachycardia (71.4% vs 27.8%; *P *= 0.04).

**Table 3:** Characteristics of the patients with and without atrial fibrillation detected by the patch-based monitoring

Characteristic	Patients with atrial fibrillation (*n *= 7)	Patients without atrial fibrillation (*n *= 36)	*P* value
Age, year	77.4 ± 6.1	68.6 ± 14.6	0.02
Male, no. (%)	4 (57.1)	22 (61.1)	1.00
Index event, no. (%)			
Stroke	5 (71.4)	30 (83.3)	0.60
TIA	2 (28.6)	6 (16.7)	
Prior stroke or TIA, no. (%)	2 (28.6)	10 (27.8)	1.00
Hypertension, no. (%)	6 (85.7)	20 (55.6)	0.22
Diabetes, no. (%)	1 (14.3)	9 (25.0)	1.00
History of ischaemic heart disease, no. (%)	4 (57.1)	4 (11.1)	0.02
Smoking status, no. (%)			
Ex-smoker	4 (57.1)	8 (22.2)	0.10
Current smoker	0 (0)	6 (16.7)	
CHADS_2_VASc_2_ score, no. (%)^a^	5.0 ± 0.6	4.3 ± 1.2	0.03
No. of days from index event to randomisation	2.3 ± 2.3	2.0 ± 1.0	0.73
Echocardiographic parameters^b^			
Left atrial enlargement^c^	6 (85.7)	11 (39.2)	0.04
Left ventricular ejection fraction (%)	53.2 ± 4.5	53.3 ± 4.3	0.94
Arrhythmia detection on patch-based monitoring			
Detection of VT	5 (71.4)	10 (27.8)	0.04
Number of episodes of VT	1.1 ± 0.9	1.3 ± 3.5	0.81
Detection of VEs	7 (100)	35 (97.2)	1.00
Percentage of total beats as VEs	3.4 ± 1.7	8.2 ± 10.5	0.44
Detection of SVEs	7 (100)	36 (100)	–
Percentage of total beats as SVEs	3.1 ± 1.8	3.0 ± 2.9	0.97
Detection of SVT	5 (83.3)	27 (75.0)	1.00
Number of SVT episodes	53.2 ± 63.2	16.4 ± 25.4	0.22

Plus–minus values are mean ± SD. *P* values were calculated with the use of Student’s *t* test, Wilcoxon Mann–Whitney Rank-Sum test or Fisher’s exact test, as appropriate. TIA denotes transient ischaemic attack

*SVE* supraventricular ectopics, *SVT* supraventricular tachycardia, *TIA* transient ischaemic attack, *VE* ventricular ectopics, *VT* ventricular tachycardia

^a^Scores on the CHADS_2_VASc_2_ risk assessment range from 0 to 6, with higher scores indicating a greater risk of stroke

^b^Transthoracic echocardiography and/or transoesophageal echocardiography was performed in 6 and 28 patients in the PAF and non-PAF groups, respectively

^c^Left atrial enlargement was defined as left atrial diameter ≥ 40 mm or left atrial volume index ≥ 29 mL/m^2^

### Economic evaluation

Implementation of the Zio Patch service at King’s College Hospital NHS Foundation Trust would result in 10.8 more strokes avoided per year compared to current practice with Holter monitoring. This would equate to a yearly saving in direct medical costs of £57,481, increasing to £106,342 over 5 years. When social care costs are included, incremental savings of £154,716 can be achieved in the first year and £410,449 at 5 years. In addition, an analysis of the potential reduction in outpatient follow-up appointment costs resulted in a further saving of £56,149, giving a total potential saving of £113,630 over the first year with the use of the ZioPatch service compared to Holter monitoring, increasing to £162,491 over 5 years.

## Discussion

In this randomised trial comparing intermediate-term ECG monitoring by means of a 14-day patch recorder with a conventional short-duration Holter monitor, monitoring with the ZioPatch resulted in a significantly higher rate of detection of PAF, with an associated greater use of anticoagulation, providing strong evidence supporting the adoption of a more prolonged monitoring approach for the detection of PAF in patients with recent cryptogenic stroke or TIA.

### Comparison with other cardiac monitoring trials

The reported incidence of PAF following cryptogenic stroke is wide-ranging from 10 to 30% [6–8]. This variation can in part be explained by differences in study design, inclusion and exclusion criteria, ECG monitoring strategies and follow-up durations. Our trial observed a 16.3% incidence of PAF in the patch-based monitoring group at 3 months, which was higher than the 8.9% incidence observed in the CRYSTAL-AF trial at 6 months [7]. This is likely because CRYSTAL-AF patients already had one round of Holter-based cardiac monitoring before inclusion. Likewise, in the EMBRACE trial, where the incidence was 11.6%, patients were recruited after having completed one Holter monitor [8]. Each round of monitoring has eliminated a proportion of PAF from the population.

A notable study difference between EPACS and the above studies is the very early cardiac monitoring, with an average of 2 days after the index stroke or TIA (compared to ~ 38 days in the CRYSTAL-AF trial and ~ 76 days in EMBRACE trial) before any cardiac monitoring. This makes our study more similar to the recent multicentre FIND-AF trial, which recruited patients within 7 days of the index event. The FIND-AF trial demonstrated that three rounds of 10-day Holter monitoring detected a PAF incidence of 14% after 6 months compared with 5% from one 24-h Holter monitor recording [15]. Three Holter ECG’s at three different time points over 6 months constitutes a fairly intensive level of cardiac monitoring and achieved comparable detection rates to a single 14-day patch-based monitor, with both being superior to short-duration Holter monitoring. Our study had significant issues in consistently delivering short-duration Holter monitoring compared to delivering patch-based monitoring suggesting that real-world implementation may favour a particular modality regardless of the duration of ECG monitoring [16]. The much earlier extended cardiac monitoring in EPACS offers the opportunity for earlier diagnosis and intervention with anticoagulation for secondary stroke prevention.

### PAF detection rate over time following a stroke

The highest yield for detecting PAF is early following a cryptogenic stroke. In the FIND-AF trial, two-thirds of the PAF events were detected within the first round of 10-day Holter monitoring rather than the subsequent two rounds [15]. In EMBRACE, half of the PAF events were detected within the first week of the 30-day monitoring period, even after patients were enrolled 2.5 months following the index ischaemic event [8]. Similarly, in CRYSTAL-AF, half of the events that were detected within the first 6 months occurred within 42 days of monitoring [7]. The results of these trials suggest that the yield of PAF detection is highest in the first few weeks to months after the ischaemic event. As a result, in addition to the extended monitoring period, the earlier device placement for the patch-based monitoring (2.1 days) compared to the short-duration Holter monitoring (38.9 days), may partially explain the higher AF detection rate observed with the patch-based strategy.

### Risk factors for PAF detection

A number of clinical features that may represent risk factors for a new diagnosis of PAF after cryptogenic stroke or TIA have been studied. We identified that patients in the patch-based monitoring group who had PAF detected were older and were more likely to have a dilated left atrium than those without PAF. Advanced age has been consistently identified as an independent risk factor [17]. An analysis of patients randomised to the implantable recorder found that increased age was the only clinical feature that was independently associated with increased incidence of PAF during follow-up [17]. There is growing evidence for the importance of premature atrial contractions and various echocardiographic parameters including left atrial enlargement [18, 19]. We found that patients with PAF were also more likely to have a history of ischaemic heart disease. Coronary artery disease is present in greater than 20% of patients with AF [20]. Whether coronary artery disease *per se* predisposes patients to AF via atrial ischaemia or whether AF interacts with coronary artery perfusion is uncertain [21]. While higher CHADS_2_/CHADS_2_VASc_2_ scores have been associated with an increased risk of AF, this parameter has not been recognised as a significant risk factor in the post-cryptogenic stroke or TIA setting [22]. Patients with PAF were more likely to have higher CHADS_2_VASc_2_ scores in our study. This is unsurprising considering the composite variables in the score are recognised individual risk factors for AF.

### Economic evaluation

While we observed no difference in the rate of recurrent TIA or stroke between the two groups, our study was not powered for this endpoint. Prolonged ECG monitoring with patch-based monitoring was predicted to prevent 10.8 more strokes than short-duration Holter monitoring. This was associated with a yearly saving in direct medical costs of £113,630, increasing to £162,491 over 5 years. Based on observed rates of AF detection and anticoagulation from the EMBRACE trial, following a recent cryptogenic stroke or TIA, both 30-day and 14-day ECG monitoring was shown to be cost-effective for preventing recurrent strokes when compared with a repeat 24-h ECG [23]. These results, as well as those from our study, lend support to emerging practice guidelines recommending longer (≥ 7 days) post-stroke ECG monitoring in patients to optimise secondary stroke prevention [4, 5, 23].

## Limitations

This study had several limitations. Firstly, the drop-out rate was approximately 20%, almost entirely due to Holter ECG service provision. With similar rates of non-compliance observed in the EMBRACE trial, compliance with Holter-based systems for extended monitoring is low, even in the context of a clinical trial. Although compliance rates for the short-duration monitoring were relatively low, the baseline characteristics were similar between the two treatment groups.

Secondly, this study did not directly compare alternative extended monitoring systems like implantable loop recorders. We chose short-duration Holter monitoring as a suitable comparator to the ZioPatch patch-based monitor to reflect current clinical practice (“gold” standard) and for real-world feasibility.

Thirdly, although it is well-established that the detection of AF following a stroke or TIA suggests that there is a casual link between stroke and previously undetected PAF, we acknowledge the possibility that the PAF detected may include the incidence of PAF in the background population as we did not study age-matched healthy controls who have not had an ischaemic stroke or TIA. Nonetheless, our incidence rates are much higher than epidemiological studies of AF and it is likely a significant proportion of the PAF we detected were causational to the index event. The REVEAL-AF trial used implantable cardiac monitors to identify an AF detection rate of 6.2% at 30 days in patients at risk of having an ischaemic stroke [24], which was lower than the incidence detected in our study.

## Conclusions

Our findings have implications for clinical practice, supporting intermediate-term ECG monitoring after a recent cryptogenic embolic stroke or TIA in patients considered appropriate potential candidates for anticoagulation. Common practice of relying on short-term monitoring for the detection of PAF after cryptogenic stroke or TIA is insufficient and should be considered as an initial screen. ZioPatch^®^ patch-based monitoring was well-tolerated and superior to standard practice of short-term ECG monitoring for the detection of PAF in patients with a cryptogenic stroke or TIA. Future, appropriately powered, studies are required to demonstrate that the increased PAF detection through extended ECG monitoring results in reduced recurrent stroke or TIA rates, thereby justifying the cost-effectiveness of using more extensive, invasive or longer-term ECG monitors.

## Additional file

**Additional file 1.** Probability and cost assumptions for economic model

## Data Availability

The datasets generated and/or analysed during the current study are not publicly available due to ethical restrictions but are available from the corresponding author on reasonable request.

## Abbreviations

AF: atrial fibrillation
ECG: electrocardiogram
EPACS: early prolonged ambulatory cardiac monitoring in stroke
CI: confidence interval
PAF: paroxysmal atrial fibrillation
SVE: supraventricular ectopics
SVT: supraventricular tachycardia
TIA: transient ischaemic attack
VE: ventricular ectopics
VT: ventricular tachycardia
