# Supplementary material for: Early prolonged ambulatory cardiac monitoring in stroke (EPACS): an open-label randomised controlled trial
Source: Eur J Med Res. 2019 Jul 26;24:25. doi: 10.1186/s40001-019-0383-8 (PMC6659210; doi:10.1186/s40001-019-0383-8)
Supplement: Supplementary file 1 — Additional file 1. Probability and cost assumptions for economic model [file 40001_2019_383_MOESM1_ESM.pdf]

Clinical inputs

Event inputs

Select Hospital:

King's College Hospital NHS Foundation Trust

	Default value	Enter alternate values below ↓	Source of default value
Ischaemic strokes - % of all strokes	87.1%	87.1%	SSNAP [1]
% of ischaemic strokes requiring outpatient monitoring	23.5%	23.5%	Putaala et al [2]
% of TIA/ outpatient treated stroke requiring outpatient monitoring	32.2%	32.2%	Amarenco et al [3]
% monitored as outpatients (any technology)	90.0%	90.0%	Assumption
Monitoring strategy (first and second pass)	1st: Holter; 2nd: repeat Holter		
% patients discharged after negative first pass monitoring	70.0%	70.0%	Assumption
% of patients receiving implantable loop after negative first/second pass	2.0%	2.0%	Assumption
% of patients receiving implantable loop after negative Zio	0.4%	0.4%	80% reduction based on user feedback
**Detection rates**			
% AF (True prevalence in target group)	30.0%	30.0%	Sanna et al [4]
% AF (Holter - first pass)	2.0%	2.0%	Teo [5]
% AF (Holter - second pass)	2.0%	2.0%	Assumption - same as first pass
% AF (5-7 day ELR - first pass)	5.7%	5.7%	Assumption: Same as second pass detection rate [6]
% AF (5-7 day ELR - second pass)	5.7%	5.7%	Jabaudon et al [6]
% AF (Zio)	16.0%	16.0%	Teo [5]
% AF (implantable monitor)	19.3%	19.3%	Weighted mean from literature (Albers et al [7])
% AF detected on chosen strategy	2.7%		Calculated
% AF detected on Zio strategy	16.1%		Calculated
Recurrent stroke in year 1 (untreated AF)	12.0%	12.0%	Hart et al [8]
Recurrent stroke in year 1 (anticoagulated AF)	4.3%	4.3%	Hart et al [8]

Event outputs

Basis of analysis Stroke + TIA

	Selected trust	
Number of in-patient strokes	1,585	HES [9]
Number of definite or probable TIA + outpatient-managed strokes	2,262	Giles & Rothwell [10].
Target patients	1,053	
Recurrent strokes attributable to AF in target patients	37.9	
Maximum preventable strokes if all patients identified and anticoagulation used	24.3	
Strokes prevented using current strategy	2.2	
Strokes prevented using Zio	13.0	
Incremental strokes avoided using Zio vs Holter	10.8	

Cost inputs and results (Medical costs only + 1 year cost horizon)

Cost inputs

Select cost horizon: 1 year

Select cost basis: Medical costs

	Default value	Enter alternate values below ↓	Source of default value
1 year medical cost of stroke	£13,452	£13,452	Xu et al [11]
1 year total cost of stroke	£22,429	£22,429	Xu et al [11]
Cost of conventional testing (per pass)	£133.43	£133.43	NHS Reference Costs [12]
Cost of Zio	£295	£295	Manufacturer
Cost of implantable monitor	£3,583	£3,583	HES [9]

Cost outputs

	Selected trust
Cost of AF-attributable strokes in target group	£509,770
Savings based on use of current monitoring strategy	£29,455
Savings based on use of Zio strategy	£175,162
Cost of current testing in target group	£181,749
Cost of implantable monitor in target group	£22,177
Cost of Zio testing in target group	£279,479
Cost of ILR in Zio group	£12,673
Cost net of testing (current strategy + ILR)	£684,241
Cost net of testing (Zio + ILR)	£626,760

Comparison of Zio vs Current strategy

Incremental cost	-£57,481
Incremental strokes prevented	10.8

Additional analysis of Out-patient opportunity costs

	Default value	Alternate value	Source
% conventionally monitored patients attending follow-up OPD	80%	80%	Assumption
% Zio monitored patients attending follow-up OPD	40%	40%	Assumption
Cost of follow-up OPD appointment	£79	£79	NHS Tariff 2017-18

	Selected trust
Number of out-patient folow-ups for selected strategy	1,090
Number of OPD follow-ups with Zio	379
Appointment saving Zio vs current strategy	711
Incremental cost Zio vs current strategy	-£56,149

Overall incremental cost using Zio **-£113,630**

Incremental cost per stroke prevented: Dominant

The analysis shows that for King's College Hospital NHS Foundation Trust the use of the Zio patch is associated with total savings of £113,630 in year 1. This means that the Zio patch has a better outcome than the current strategy at a lower overall cost

Cost inputs and results (Medical costs only + 5 year cost horizon)

Cost inputs

Select cost horizon	5 year
Select cost basis	Medical costs

	Default value	Enter alternate values below ↓	Source of default value
5 year medical cost of stroke	£17,963	£17,963	Xu et al [11]
5 year total cost of stroke	£46,039	£46,039	Xu et al [11]
Cost of conventional testing (per pass)	£133.43	£133.43	NHS Reference Costs [12]
Cost of Zio	£295	£295	Manufacturer
Cost of implantable monitor	£3,583	£3,583	HES [9]

Cost outputs

	Selected trust
Cost of AF-attributable strokes in target group	£680,716
Savings based on use of current monitoring strategy	£39,333
Savings based on use of Zio strategy	£233,900
Cost of current testing in target group	£181,749
Cost of implantable monitor in target group	£22,177
Cost of Zio testing in target group	£279,479
Cost of ILR in Zio group	£12,673
Cost net of testing (current strategy + ILR)	£845,309
Cost net of testing (Zio + ILR)	£738,967
**Comparison of Zio vs Current strategy**	
Incremental cost	-£106,342
Incremental strokes prevented	10.8

Additional analysis of Out-patient opportunity costs

	Default value	Alternate value	Source
% conventionally monitored patients attending follow-up OPD	80%	80%	Assumption
% Zio monitored patients attending follow-up OPD	40%	40%	Assumption
Cost of follow-up OPD appointment	£79	£79	NHS Tariff 2017-18

	Selected trust
Number of out-patient folow-ups for selected strategy	1,090
Number of OPD follow-ups with Zio	379
Appointment saving Zio vs current strategy	711
Incremental cost Zio vs current strategy	-£56,149
**Overall incremental cost using Zio**	**-£162,491**

Incremental cost per stroke prevented: Dominant

The analysis shows that for King's College Hospital NHS Foundation Trust the use of the Zio patch is associated with total savings of £162,491 over 5 years. This means that the Zio patch has a better outcome than the current strategy at a lower overall cost

Cost inputs and results (Medical & Social Care costs + 1 year cost horizon)

Cost inputs

Select cost horizon	1 year
Select cost basis	Medical + Social Care costs

	Default value	Enter alternate values below ↓	Source of default value
1 year medical cost of stroke	£13,452	£13,452	Xu et al [11]
1 year total cost of stroke	£22,429	£22,429	Xu et al [11]
Cost of conventional testing (per pass)	£133.43	£133.43	NHS Reference Costs [12]
Cost of Zio	£295	£295	Manufacturer
Cost of implantable monitor	£3,583	£3,583	HES [9]

Cost outputs

	Selected trust
Cost of AF-attributable strokes in target group	£849,957
Savings based on use of current monitoring strategy	£49,112
Savings based on use of Zio strategy	£292,053
Cost of current testing in target group	£181,749
Cost of implantable monitor in target group	£22,177
Cost of Zio testing in target group	£279,479
Cost of ILR in Zio group	£12,673
Cost net of testing (current strategy + ILR)	£1,004,772
Cost net of testing (Zio + ILR)	£850,056
**Comparison of Zio vs Current strategy**	
Incremental cost	-£154,716
Incremental strokes prevented	10.8

Additional analysis of Out-patient opportunity costs

	Default value	Alternate value	Source
% conventionally monitored patients attending follow-up OPD	80%	80%	Assumption
% Zio monitored patients attending follow-up OPD	40%	40%	Assumption
Cost of follow-up OPD appointment	£79	£79	NHS Tariff 2017-18

	Selected trust
Number of out-patient folow-ups for selected strategy	1,090
Number of OPD follow-ups with Zio	379
Appointment saving Zio vs current strategy	711
Incremental cost Zio vs current strategy	-£56,149
**Overall incremental cost using Zio**	**-£210,865**

Incremental cost per stroke prevented: Dominant

The analysis shows that for King's College Hospital NHS Foundation Trust the use of the Zio patch is associated with total savings of £210,865 in year 1. This means that the Zio patch has a better outcome than the current strategy at a lower overall cost

Cost inputs and results (Medical & Social Care costs + 5 year cost horizon)

Cost inputs

Select cost horizon: 5 year

Select cost basis: Medical + Social Care costs

	Default value	Enter alternate values below ↓	Source of default value
5 year medical cost of stroke	£17,963	£17,963	Xu et al [11]
5 year total cost of stroke	£46,039	£46,039	Xu et al [11]
Cost of conventional testing (per pass)	£133.43	£133.43	NHS Reference Costs [12]
Cost of Zio	£295	£295	Manufacturer
Cost of implantable monitor	£3,583	£3,583	HES [9]

Cost outputs

	Selected trust
Cost of AF-attributable strokes in target group	£1,744,669
Savings based on use of current monitoring strategy	£100,810
Savings based on use of Zio strategy	£599,484
Cost of current testing in target group	£181,749
Cost of implantable monitor in target group	£22,177
Cost of Zio testing in target group	£279,479
Cost of ILR in Zio group	£12,673
Cost net of testing (current strategy + ILR)	£1,847,785
Cost net of testing (Zio + ILR)	£1,437,336
**Comparison of Zio vs Current strategy**	
Incremental cost	-£410,449
Incremental strokes prevented	10.8

Additional analysis of Out-patient opportunity costs

	Default value	Alternate value	Source
% conventionally monitored patients attending follow-up OPD	80%	80%	Assumption
% Zio monitored patients attending follow-up OPD	40%	40%	Assumption
Cost of follow-up OPD appointment	£79	£79	NHS Tariff 2017-18

	Selected trust
Number of out-patient folow-ups for selected strategy	1,090
Number of OPD follow-ups with Zio	379
Appointment saving Zio vs current strategy	711
Incremental cost Zio vs current strategy	-£56,149
**Overall incremental cost using Zio**	**-£466,598**

Incremental cost per stroke prevented: Dominant

The analysis shows that for King's College Hospital NHS Foundation Trust the use of the Zio patch is associated with total savings of £466,598 over 5 years. This means that the Zio patch has a better outcome than the current strategy at a lower overall cost

References

1. Stroke Sentinel National Audit Programme (SSNAP). National results (Clinical) April 2016-March 2017. Available at: https://www.strokeaudit.org/results/Clinical-audit/National-Results.aspx
2. Putaala J et al. Undetermined stroke with an embolic pattern. A common phenotype with high early recurrence risk. Ann Med 2015;47:406-13
3. Amarenco P et al. One-year risk of stroke after transient ischaemic attack or minor stroke. N Engl J Med 2016;374:1533-42
4. Sanna T et al. Cryptogenic stroke and underlying atrial fibrillation. N Engl J Med 2014;370:2478-86
5. Teo J. Early prolonged ambulatory cardiac monitoring in stroke (EPACS) - open label randomised clinical trial. Presented at: 3rd European Stroke Conference. Prague 16-18 May 2017
6. Jabaudon D et al. Usefulness of ambulatory 7-day ECG monitoring for the detection of atrial fibrillation and flutter after acute stroke and transient ischaemic attack. Stroke 2004;35:1647-51.
7. Albers G et al. Heart rhythm monitoring strategies for cryptogenic stroke: 2015 diagnostics and monitoring stroke focus group report. J Am Heart Assoc 2016;5:e002944
8. Hart R et al. Meta-analysis: antithrombotic therapy to prevent stroke in patients who have nonvalvular atrial fibrillation. Ann Intern med 2007;146:857-67.
9. Harvey-Walsh. Bespoke analysis of Hospital Episode Statistics for England (Oct 2016-Sept 2017)
10. Giles M, Rothwell P. Substantial underestimation of the need fro outpatient services for TIA and minor stroke. Age Ageing 2007;36:676-80.
11. Xu X-M et al. The economic burden of stroke care in England, Wales and Northern Ireland: Using a national stroke register to estimate and report patient-level health economic outcomes in stroke. Eur Stroke J 2018;3:82-91
12. NHS Improvement. NHS Reference Costs 2016-17. Available at: https://improvement.nhs.uk/documents/1973/2_-_National_schedule_of_reference_costs_-_the_main_schedule.xlsx
